# Sodium‐glucose cotransporter 2 inhibition does not reduce hepatic steatosis in overweight, insulin‐resistant patients without type 2 diabetes

**DOI:** 10.1002/jgh3.12274

**Published:** 2019-11-05

**Authors:** Thomas Marjot, Charlotte J Green, Catriona A Charlton, Thomas Cornfield, Jonathan Hazlehurst, Ahmad Moolla, Sarah White, Jane Francis, Stefan Neubauer, Jeremy FL Cobbold, Leanne Hodson, Jeremy W Tomlinson

**Affiliations:** ^1^ Translational Gastroenterology Unit, NIHR Oxford Biomedical Research Centre University of Oxford, John Radcliffe Hospital Oxford UK; ^2^ Oxford Centre for Diabetes, Endocrinology and Metabolism, NIHR Oxford Biomedical Research Centre University of Oxford, Churchill Hospital Oxford UK; ^3^ Institute of Metabolism and Systems Research University of Birmingham Birmingham UK; ^4^ Centre of Endocrinology, Diabetes and Metabolism Queen Elizabeth Hospital Birmingham, Birmingham Health Partners Birmingham UK; ^5^ Oxford Centre for Clinical Magnetic Resonance Research (OCMR), Division of Cardiovascular Medicine, Radcliffe Department of Medicine University of Oxford Oxford UK

**Keywords:** fatty liver, lipogenesis, magnetic resonance spectroscopy, sodium‐glucose transporter 2

## Abstract

**Background and Aim:**

Non‐alcoholic fatty liver disease (NAFLD) is rapidly becoming the leading indication for liver transplant and is associated with increased cardiovascular and liver mortality, yet there are no licensed therapies. Sodium‐glucose cotransporter 2 (SGLT2) inhibitors are widely used for their glucose‐lowering effects in patients with type 2 diabetes (T2D). Preclinical models have suggested a beneficial impact on NAFLD, but clinical data are limited, and there are currently no data on patients without T2D. We aimed to investigate the impact of SGLT2 inhibition on NAFLD in overweight, nondiabetic patients and establish the effect these agents may have on the processes that regulate hepatic steatosis in vivo.

**Methods:**

We conducted an open‐label, experimental medicine pilot study on insulin‐resistant overweight/obese individuals (*n* = 10) using gold‐standard noninvasive assessments of NAFLD phenotype, including magnetic resonance spectroscopy, two‐step hyperinsulinemic euglycemic clamps, and stable isotope tracers to assess lipid and glucose metabolism. Investigations were performed before and after a 12‐week treatment with the SGLT2 inhibitor, dapagliflozin.

**Results:**

Despite a body weight reduction of 4.4 kg, hepatic steatosis was unchanged following treatment. Hepatic glucose production increased, and there was impairment of glucose disposal during the low‐dose insulin infusion. Although circulating, nonesterified, fatty acid levels did not change, the ability of insulin to suppress lipolysis was reduced.

**Conclusions:**

SGLT2 inhibition for 12 weeks does not improve hepatic steatosis in patients without T2D. Additional studies in patients with established T2D or impairments of fasting or postprandial glucose homeostasis are needed to determine whether SGLT2 inhibition represents a viable therapeutic strategy for NAFLD. (http://clinicaltrials.gov Number NCT02696941).

## Graphical abstract

Sodium‐glucose cotransporter 2 (SGLT2) inhibitors have been shown to improve hepatic steatosis in patients with non‐alcoholic fatty liver disease (NAFLD) and type 2 diabetes, but data in patients without diabetes are lacking. In a small experimental medicine study, we show that, despite a reduction in body weight, SGLT2 inhibition does not alter hepatic steatosis in overweight human participants without diabetes. De novo lipogenesis and circulating nonesterified fatty acids, which are both upregulated in NAFLD, were unaffected by SGLT2 inhibition. It seems unlikely that SGLT2 inhibition will be of significant benefit to patients with NAFLD who have normoglycemia.

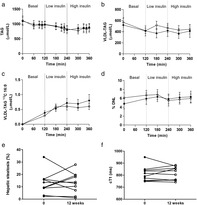

## Introduction

Non‐alcoholic fatty liver disease (NAFLD) has reached epidemic proportions affecting up to 30% of the general population and 70–90% of individuals with type 2 diabetes (T2D) and/or obesity.^1^, ^2^ It drives increased morbidity and mortality, both through a specific impact on the liver and through adverse cardiovascular outcomes^3^; by 2020, NAFLD will become the leading indication for liver transplantation worldwide.^4^ NAFLD is a spectrum of disease ranging from simple steatosis to inflammation (non‐alcoholic steatohepatitis [NASH]), cirrhosis, and hepatocellular carcinoma (HCC). Despite the burden of disease, there are currently no licensed treatments for NAFLD.

While the development of diabetes is a major driver for NAFLD progression,^5^ up to 75% of those with NAFLD will not have T2D.^6^ A crucial component of diabetes and NAFLD risk is both hepatic and peripheral insulin resistance. Using hyperinsulinemic euglycemic clamp techniques incorporating the use of stable isotope infusions, studies have identified the liver (with increased glucose production), muscle (with decreased glucose disposal), and adipose tissue (failure of insulin to suppress lipolysis) as important sites of insulin resistance in patients with NAFLD.^7^

The processes that govern hepatic triacylglycerol (TAG) accumulation are complex and represent a balance between synthesis and utilization. Nonesterified fatty acids (NEFAs), largely from adipose tissue, are delivered to the liver via the portal circulation and are esterified to form TAG. While this is the largest contributor to the hepatic TAG pool, the synthesis of fatty acids from nonlipid precursors (most commonly glucose) in the process of de novo lipogenesis (DNL) becomes increasingly important in established NAFLD.^8^, ^9^ Based on these observations, there is significant clinical interest in targeting DNL as a potential therapeutic strategy in the treatment of NAFLD.^10^

The sodium‐glucose cotransporter type 2 (SGLT2) inhibitors are a recently licensed class of antidiabetic agents that promote glucosuria through decreased renal glucose reabsorption, which in turn improves insulin sensitivity and glycemic control.^11^, ^12^ In addition, the EMPA‐REG study has demonstrated the positive impact that these agents may have on cardiovascular outcomes.^13^ SGLT2 inhibitors have been shown to be effective in several rodent models of NAFLD. Using a choline‐deficient diet to recapitulate some of the histological features of the NAFLD while maintaining normoglycemia, 5 weeks of SGLT2 inhibition led to significant reductions in hepatic TAG content and improved markers of liver fibrosis.^14^ Similarly, 4 weeks of treatment in obese mice with T2D reduced hepatic steatosis and markers of liver oxidative stress.^15^ Data from human studies are more limited, but recent randomized control studies have demonstrated that SGLT2 inhibitors reduce liver fat and improve liver function tests in patients with T2D and NAFLD.^16^, ^17^, ^18^, ^19^ A small uncontrolled prospective study has suggested that SGLT2 inhibition may improve liver histology in NAFLD patients with T2D.^20^

Studies investigating the impact of glucose‐lowering therapies to treat NAFLD are often challenging to interpret due to confounding factors that include the use of additional medications (e.g. insulin, thiazolidinediones [TZDs], glucagon‐like peptide‐1 [GLP‐1] analogues, or dipeptidyl peptidase 4 [DPP‐4] inhibitors) and changes in weight alongside improvements in glycemic control in patients with T2D. To address some of these issues, we have performed an experimental medicine study using gold‐standard noninvasive techniques to assess the impact of SGLT2 inhibition in treatment‐naïve, overweight, and obese individuals without T2D. Our aim was to determine if SGLT2 inhibition might represent a valid therapeutic strategy in these individuals, as well as identify the mechanisms by which these agents might be acting to regulate hepatic steatosis in vivo.

## Methods

### *Study design and participants*

We performed a single‐center, open‐label trial of 12 weeks of therapy using the SGLT2 inhibitor dapagliflozin at 10 mg administered once daily in treatment‐naïve, insulin‐resistant, overweight/obese patients at risk of NAFLD. Participants were aged 18–70 years; had a body mass index (BMI) between 25 and 45 kg/m^2^; did not have T2D (HbA1C 32–48 mmol/mol); and were not taking medications known to alter hepatic, blood glucose, or weight. Participants consuming excess alcohol (females >14 units/week and males >21 units/week) or with comorbid liver disease were excluded. Patients were excluded if they self‐reported significant attempts to lose weight within the last 3 months or if they had enrolled in any medically or commercially available weight reduction programs. The clinical study protocol received full ethical approval from the North of Scotland Research Ethics Committee (REC ref. 15/NS/0117) and local institutional review board. All adult subjects gave informed written consent prior to participation.

### *Clinical protocol*

Baseline investigations included the assessment of hepatic TAG content using magnetic resonance spectroscopy (MRS) and a two‐step hyperinsulinemic euglycemic clamp incorporating the use of stable isotope tracers. Imaging and metabolic assessments were performed within 1 week, after which time, treatment with 10 mg dapagliflozin once daily was started. All investigations were repeated after 12 weeks of treatment, after which all treatment was stopped. Metabolic equivalent of task (MET) minutes of exercise were calculated for the week before baseline and follow‐up investigations using the international physical activity questionnaire (IPAQ). Average daily energy requirements were also calculated for the week before baseline and follow‐up investigations using data from a food diary self‐completed by participants. All authors had access to the study data and reviewed and approved the final manuscript.

### *Magnetic resonance protocol*

Hepatic steatosis was quantified using localized cardiac‐triggered proton spectroscopy.^21^ NAFLD was defined by steatosis >5.6%, which represents the 95th percentile of hepatic TAG content across the general population.^22^ Hepatic water and iron content were quantified using T1 and T2* mapping, respectively, and an “iron‐corrected T1” (cT1) calculated as surrogate marker of inflammation and fibrosis.^21^

### *De novo lipogenesis*

Participants were provided with deuterated water (^2^H_2_O) to drink the evening before and throughout the two‐step hyperinsulinemic euglycemic clamp. Incorporation of ^2^H from the ^2^H_2_O into very low‐density lipoprotein (VLDL) TAG‐palmitate was used to determine hepatic DNL.

### *Two‐step hyperinsulinemic euglycemic clamp*

Arterialized blood was sampled to determine the blood glucose concentration to be maintained (clamped) throughout the study. Infusions of U‐2,2 D2‐glucose, and [U^13^C]palmitic acid (to label the plasma NEFA pool) were then commenced, and steady‐state blood samples were taken after 2 h of this “basal phase.” Two phases of insulin were then infused: 2 h at “low dose” (20 mU/m^2^/min) and then 2 h at “high‐dose” (100 mU/m^2^/min). The rate of a simultaneous infusion of 20% glucose enriched with 2,2 D2‐glucose was titrated at 5‐min intervals to maintain fasting glycemic levels. Steady‐state blood was sampled at the end of each phase. Rates of hepatic endogenous glucose production (EGP) and glucose disposal (Gd) were calculated using modified versions of the Steele Equations.^23^

### *Fatty acid oxidation*

Breath samples were collected at half hourly intervals throughout the clamp, and the generation of ^13^CO_2_ (from the infused ^13^C‐palmitate) was measured as a reflection of global fatty acid oxidation.

## Results

Ten patients (five male and five female) were enrolled in the study. The mean age was 50.8 ± 0.8 years, and all were either overweight or obese with a mean baseline BMI of 32.8 ± 1.2 kg/m^2^. At baseline, 8 of 10 participants had non‐alcoholic fatty liver as defined by >5.6% steatosis on MRS. All participants completed the study protocol, and no significant adverse events were reported.

### *Body composition and anthropometry*

Mean total body weight decreased by 4.5 ± 1.4 kg (−4.63%, *P* < 0.05) due to a reduction in both fat mass (0.9 ± 2.8 kg, NS) and lean mass (2.3 ± 2.1 kg, *P* < 0.01). Total body water also decreased by 1.6 ± 1.1 kg (*P* < 0.05). Systolic blood pressure reduced by 6 ± 7 mmHg (*P* = 0.57), and diastolic blood increased by 4 ± 2 mmHg (*P* = 0.31) Waist circumference remained unchanged. There were no significant differences in self‐reported calorific intake and exercise levels in the week preceding baseline and follow‐up investigations as measured by a 7‐day food diary and short‐form IPAQ, respectively (Table 1).

**Table 1:** Study characteristics, magnetic resonance parameters, and fasting plasma biochemistry in 10 insulin‐resistant, obese/overweight individuals before and after 12 weeks of SGLT2 inhibition with dapagliflozin 10 mg once daily

	Pre‐dapagliflozin	Post‐dapagliflozin
Subject characteristics		
Gender (F/M)	5/5	5/5
Age (years)	50.8 ± 0.8	50.8 ± 0.8
Weight (kg)	95.1 ± 4.2	90.7 ± 3.9*
BMI (kg/m^2^)	32.8 ± 1.2	31.3 ± 1.3**
Body fat (%)	35.1 ± 2.9	34.2 ± 3.2
Absolute fat mass (kg)	33.8 ± 10.3	31.3 ± 10.9*
Total body water (kg)	44.9 ± 8.7	43.3 ± 7.6*
Lean mass (kg)	61.6 ± 3.7	59.3 ± 3.3**
HbA1c (mmol/mol)	39.3 ± 2.5	36.9 ± 1.7
Blood pressure (mmHg)	135 ± 7(systolic)	129 ± 6 (systolic)
	77 ± 3 (diastolic)	81 ± 2(diastolic)
Waist circumference (cm)	107.0 ± 3.5	107.7 ± 4
MET minutes of exercise a week	35 208 ± 2895.7	5289.7 ± 2570
Average daily energy consumption (kcal)	2558.3 ± 318	2698 ± 308
Magnetic resonance imaging		
Hepatic steatosis (%)	12.9 ± 2.9	13.2 ± 2.7
Intrahepatic iron (mg/g)	1.0 ± 0.04	1.0 ± 0.04
Iron‐corrected T1 (cT1) (ms)	811.2 ± 21.9	813.1 ± 19.5
Fasting plasma biochemistry		
ALT (U/L)	35.0 ± 4.8	27.3 ± 1.9
Glucose (mmol/L)	5.7 ± 0.2	5.4 ± 0.1
NEFA (μmol/L)	624 ± 79	613 ± 76
Glycerol (mU/L)	58.7 ± 12.0	53.6 ± 9.4
Triglyceride (mmol/L)	1.1 ± 0.1	1.1 ± 0.2
3OHB (μmol/L)	141.1 ± 44.7	151.8 ± 25.3
Urea (mmol/L)	5.2 ± 0.4	5.6 ± 0.3
ApoB (g/L)	0.93 ± 0.05	0.93 ± 0.06
Total cholesterol (mmol/L)	4.8 ± 0.3	4.7 ± 0.3
HDL cholesterol (mmol/L)	1.2 ± 0.1	1.2 ± 0.1
Insulin (mU/L)	23.7 ± 2.8	21.0 ± 2.3*
HOMA‐IR	5.1 ± 0.7	4.1 ± 0.5*

* *P* < 0.05,

** *P* < 0.01 pre‐ *versus* post‐dapagliflozin.

Data are presented as mean ± standard error.

3OHB, 3‐hydroxy‐butyrate; ALT, alanine aminotransferase; ApoB, apolipoprotein B; BMI, body mass index; HbA1c, hemoglobin A1c; HDL, high‐density lipoprotein; HOMA‐IR, homeostatic model assessment of insulin resistance; MET, metabolic equivalent of task; NEFA, nonesterified fatty acid.

### *Peripheral insulin sensitivity*

**Figure 1:**
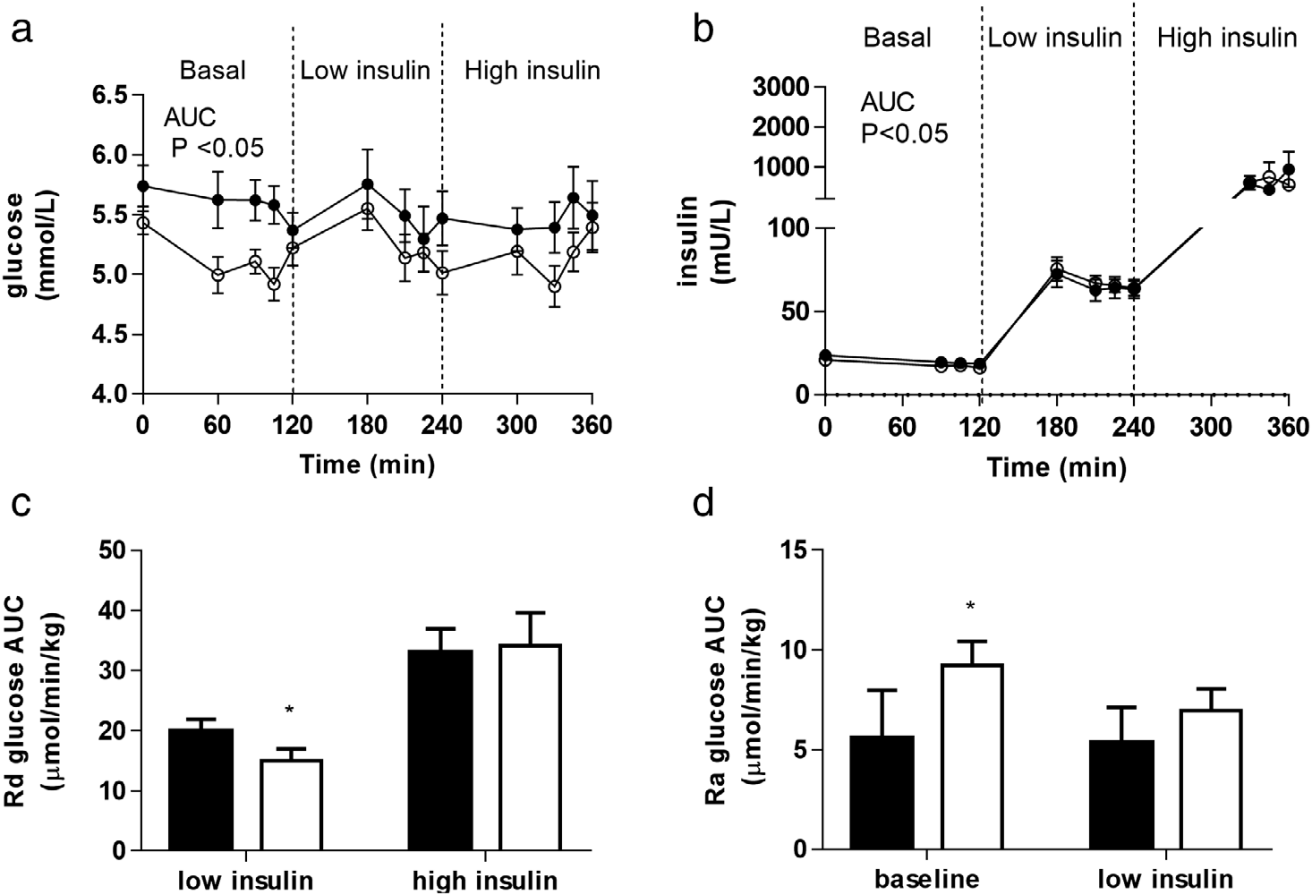
Plasma glucose (a) and plasma insulin (b) levels across a two‐step hyperinsulinemic euglycemic clamp before (filled circles) and after (open circles) 12 weeks of treatment with the sodium‐glucose cotransporter 2 inhibitor, dapagliflozin. Glucose disposal (Rd glucose) during the low‐dose insulin infusion decreased following dapagliflozin treatment but was unchanged during high‐dose insulin (c). Basal glucose production rates (Ra glucose) increased after 12 weeks of dapagliflozin treatment but were not different during low‐dose insulin infusion (d) (data presented are mean ± standard error of mean; *P < 0.05, pre‐dapagliflozin = black bars, post‐dapagliflozin = open bars). AUC, area under curve.

Although fasting glucose levels were unchanged following 12 weeks of treatment, fasting insulin levels decreased, as did the homeostatic model assessment of insulin resistance (HOMA‐IR) (Table 1). Glucose and insulin levels across the clamp were the same before and after treatment (Fig. 1a,b). Glucose disposal (Rd glucose) following low‐dose insulin infusion decreased following treatment indicative of impaired insulin action. However, after high‐dose insulin, there was no difference in Rd glucose before or after treatment (Fig. 1c).

### *Hepatic insulin sensitivity and TAG content*

Alanine aminotransferase (ALT) decreased, but this did not reach statistical significance (*P* = 0.06) (Table 1). Basal glucose production rate (Ra glucose) increased (5.6 ± 2.4 *vs* 9.2 ± 1.2 μmol/min/kg, *P* < 0.05), but there was no difference in the ability of low‐dose insulin to suppress Ra glucose (Fig. 1d).

**Figure 2:**
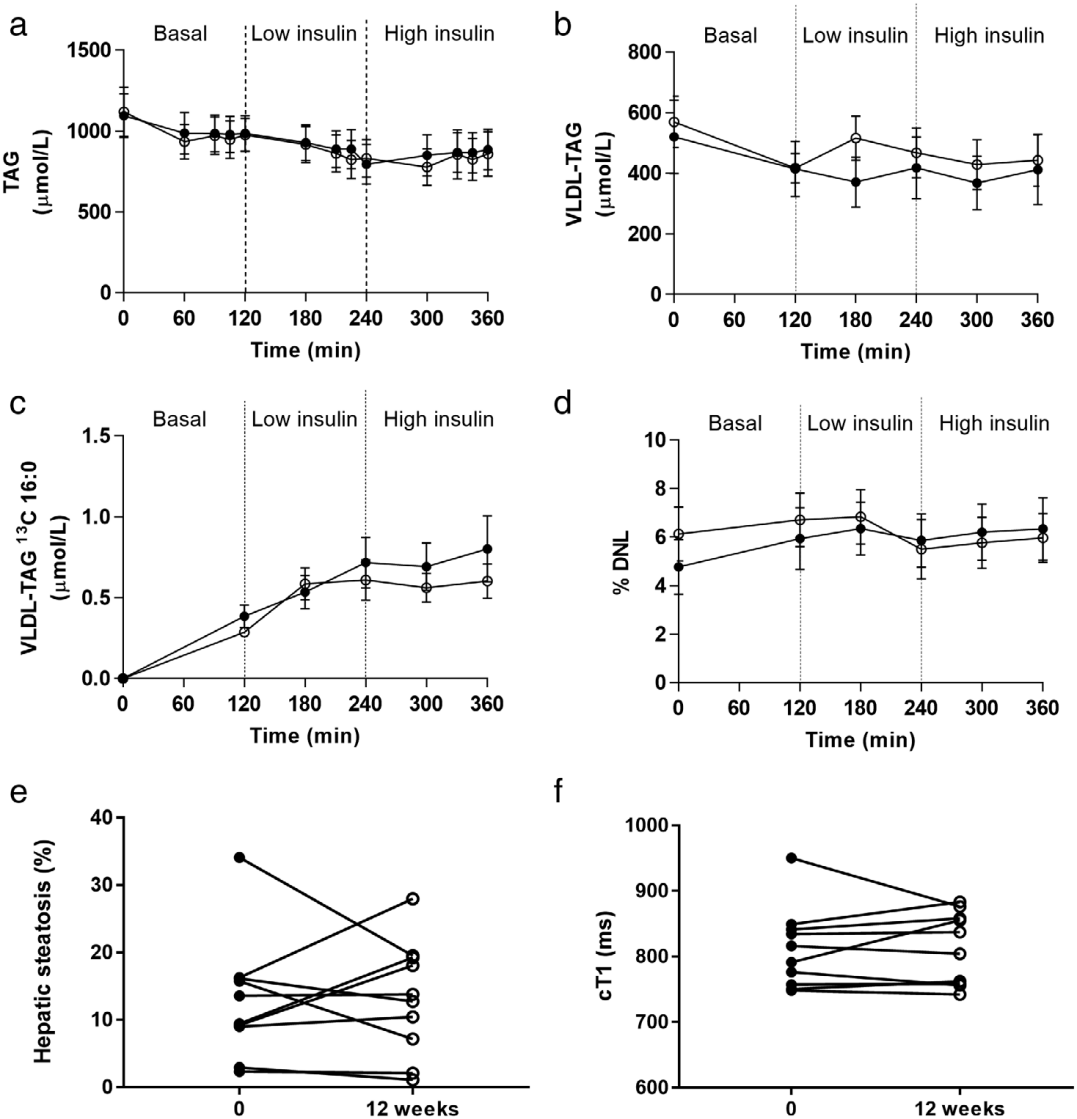
Plasma total TAG (a) and VLDL TAG (b) decreased across the hyperinsulinemic clamp (both *P* < 0.001 effect of time). There was no impact of treatment with dapagliflozin. The incorporation of ^13^C‐palmitate into VLDL‐TAG (c) and ^2^H from ^2^H_2_O into palmitate (d) did not change. Hepatic TAG content as measured by magnetic resonance spectroscopy (e) and cT1 values (f) did not change with dapagliflozin treatment. Data presented are mean ± standard error of mean; pre‐dapagliflozin = filled circles/bars, post‐dapagliflozin = open circles/bars. DNL, de novo lipogenesis; TAG, triacylglycerol; VLDL, very low‐density lipoprotein.

**Figure 3:**
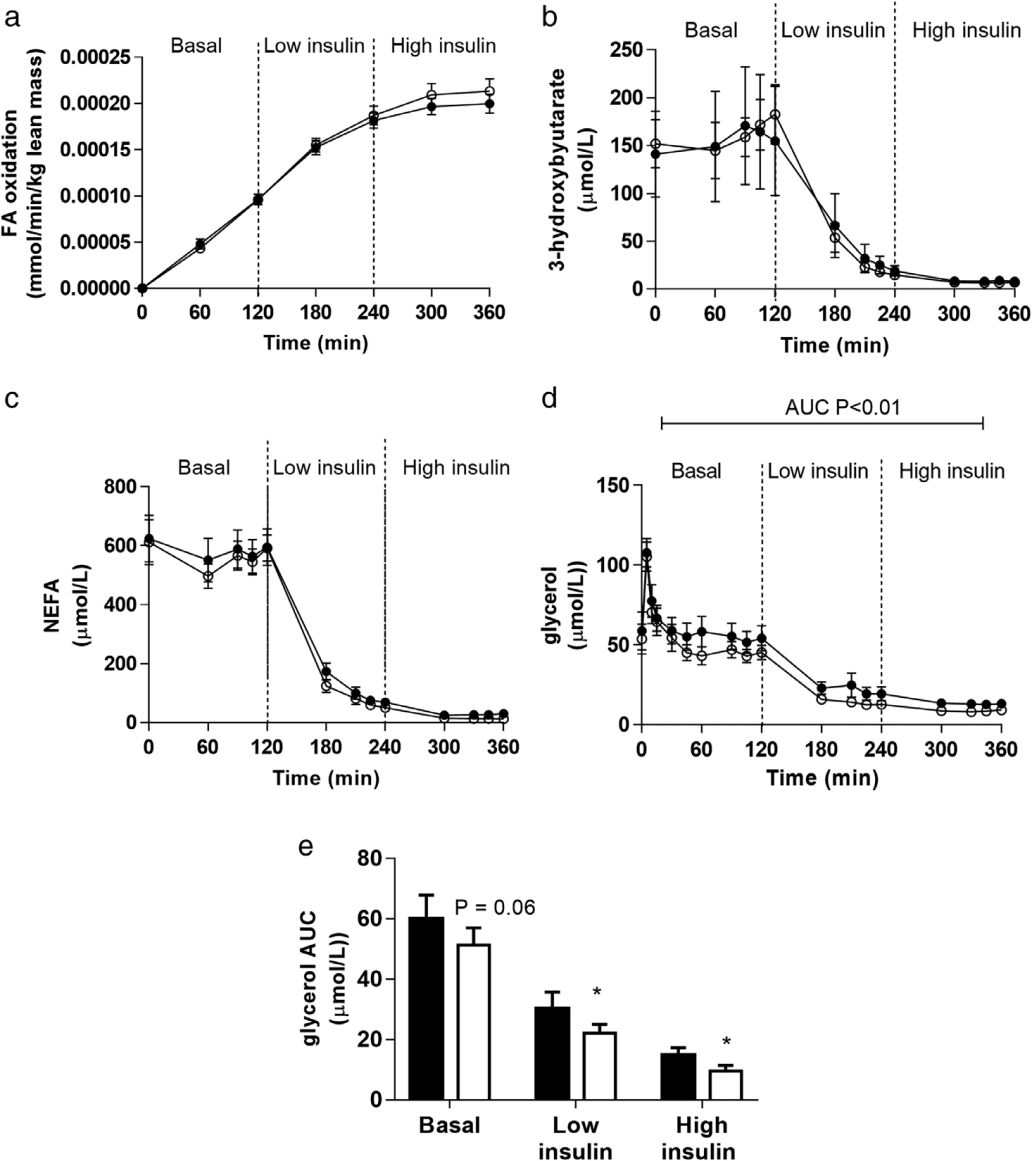
Whole‐body fatty acid oxidation as measured by ^13^CO_2_ in breath samples from infused ^13^C‐palmitate (a), hepatic fatty acid oxidation determined by 3‐hydroxybutyrate (b), and plasma NEFA levels (c) were unaltered by treatment with dapagliflozin. While fasting plasma glycerol levels were the same pre‐ and post‐dapagliflozin treatment, there was a more pronounced suppression of glycerol during the basal and low‐ and high‐dose insulin infusions after dapagliflozin treatment (d and e). Data presented are mean ± standard error of mean; pre‐dapagliflozin = filled circles/bars, post‐dapagliflozin = open circles/bars. AUC, area under curve; FA, fatty acid; NEFA, nonesterified fatty acid.

There were no differences in fasting total circulating TAG or VLDL‐TAG before or after treatment. Both decreased across the clamp, and this suppression was unaffected by dapagliflozin (Fig. 2a,b). Incorporation of ^13^C‐palmitate into VLDL‐TAG reflecting re‐esterification was not different before or after treatment (Fig. 3c). Incorporation of ^2^H in VLDL‐TAG‐palmitate (reflecting the contribution of DNL to the hepatic lipid pool) was also unaltered by dapagliflozin (Fig. 2d).

### *Magnetic resonance imaging and spectroscopy*

Mean fat fraction at baseline was 12.9 ± 2.9% compared with 13.2 ± 2.7% after 12 weeks of treatment (*P* = 0.7). Mean hepatic iron content (1 ± 0.04 *vs* 1 ± 0.04 mg/g) and whole liver cT1 values (811.2 ± 21.9 *vs* 813.1 ± 19.5 ms, *P* = 0.9) remained unchanged pre‐ and posttreatment (Fig. 2e,f).

### *Fatty acid oxidation*

Total body oxidation of fatty acid was measured by the appearance of ^13^CO_2_ in the breath, and this increased over time. There was no difference in ^13^CO_2_ appearance rate before or after dapagliflozin treatment (Fig. 3a). 3‐hydroxy‐butyrate (3OHB) is an end‐product of fatty acid oxidation. As expected, insulin suppressed 3OHB generation across the clamp, but this was unaffected by dapagliflozin (Fig. 3b).

### *Circulating NEFA and glycerol*

Fasting circulating NEFA levels were not different before and after dapagliflozin treatment (Table 1). In addition, dapagliflozin did not alter the ability of insulin to suppress circulating NEFA levels during the hyperinsulinemic clamp (Fig. 3c). However, although fasting glycerol levels were not different before and after treatment, across the clamp, there was enhanced insulin‐mediated suppression of glycerol after dapagliflozin treatment, potentially reflecting enhanced adipose tissue insulin sensitivity [glycerol area under curve (AUC): low insulin 30.3 ± 5.5 *vs* 22.2 ± 2.9 mU/L.h, *P* < 0.06; high insulin 14.7 ± 2.2 *vs* 9.5 ± 1.9 mU/L.h, *P* < 0.05, before *vs* after Dapagliflozin] (Fig. 3d,e).

A subgroup analysis was performed excluding the two participants without clinically significant hepatic steatosis (<5.6% on MRS). The significant findings were unaltered by their exclusion.

## Discussion

Using an experimental medicine approach incorporating currently accepted gold‐standard noninvasive metabolic assessments in patients with NAFLD, we have assessed the impact of SGLT2 inhibition using dapagliflozin on the tissue‐specific insulin‐regulated processes that drive the accumulation of TAG within the liver. This is the first study to evaluate the effect of SGLT2 inhibition on NAFLD in patients without T2D and to assess hepatic steatosis using MRS.

We have shown that 12 weeks of treatment with dapagliflozin did not alter hepatic steatosis on MRS. In agreement with these data, we did not observe any significant change in the contribution of DNL to VLDL‐TAG, suggesting that there would be no change in the contribution of DNL to hepatic TAG accumulation. Furthermore, we found no change in global measures of lipid oxidation or any beneficial impact on hepatic insulin sensitivity. While we did observe enhanced suppression of glycerol following insulin infusion, there were no significant changes in NEFA, and therefore, it is likely that NEFA delivery to the liver with subsequent re‐esterification to TAG would be unaltered. The apparent lack of effect on lipolysis in our study contrasts with data in patients with T2D where SGLT2 inhibition increased circulating NEFA and beta‐hydroxybutyrate.^18^ It has been postulated that increased lipolysis with SGLT2 inhibitors may be mediated through increased glucagon secretion,^24^ although circulating glucagon levels were not measured in the current study.

The evidence base for the potential benefits of SGLT2 inhibition in the context of NAFLD treatment is growing. Recent randomized control data from the E‐LIFT trial demonstrated reduced hepatic steatosis on MRS with empagliflozin.^16^ Several groups have also recently demonstrated a benefit on liver fat fraction with dapagliflozin and canagliflozin.^17^, ^18^, ^19^ However, another human study, which included an exploratory subgroup analysis using magnetic resonance imaging to look at changes in hepatic steatosis in patients with poorly controlled T2D, did not identify a significant effect.^25^ All of these data, however, are derived from patients with comorbid T2D and NAFLD, with no dedicated studies, to our knowledge, conducted on NAFLD patients without T2D.

Although not statistically significant, ALT fell by 20% in the current study. This is in line with data from randomized trials of SGLT2 inhibitors in T2DM, which demonstrate similar reductions in liver enzymes independent of changes in weight and HbA1c.^26^ This may suggest a liver‐specific effect of SGLT2 inhibitors, although ALT has been shown to be a poor biomarker for hepatic steatosis.^27^

There are several studies investigating SGLT2 inhibitors in NAFLD that are actively recruiting (https://clinicaltrials.gov/ct2/home), although all are exclusively on patients with T2D. While rodents provide compelling evidence, including in NAFLD models with normoglycaemia,^14^, ^15^ there are limitations with the use of rodent models,^28^ and therefore, undertaking detailed mechanistic studies using treatments that have been shown to be safe and well tolerated is warranted.

Glucose is the major substrate for DNL in patients with T2D. Coexistent hepatic and global (skeletal muscle and adipose) insulin resistance results in unchecked NEFA delivery to the liver and, when combined with the impact on DNL, drives hepatic TAG accumulation. In patients with T2D, SGLT2 inhibitors, through their glycosuric effect of lowering blood glucose, would therefore potentially decrease glucose delivery to the liver and limit DNL. In addition, any impact to enhance insulin sensitivity (and to promote weight loss) would decrease NEFA mobilization and delivery to the liver for re‐esterification. However, it is important to note that up to 75% of patients with NAFLD do not have T2D^6^ and therefore would not have access to medications licensed for the treatment of T2D, which have been shown to have histological benefit in NAFLD.^29^

There are perhaps several explanations regarding the lack of effect that we observed in our patients. Importantly, our patients did not have T2D, and we did not observe any change in blood glucose levels; this may be fundamental in understanding the lack of change in DNL. In patients with T2D, 2 weeks of treatment with SGLT2 inhibition enhanced peripheral glucose disposal but also increased EGP rate.^30^ While it has been proposed that this may reflect the amelioration of glucotoxicity as a consequence of renal glucose loss and the consequent improvement in glycemic control, the precise mechanisms underpinning this observation remain to be determined. Recent evidence has also suggested that SGLT2 inhibition can augment the actions of GLP,^31^ and this mechanism may be important when considering any potential benefit to patients with NAFLD. In our study, we did observe a significant increase in Ra glucose in the basal state following SGLT2 inhibition, although suppression by low‐dose insulin was not changed. Contrary to findings in patients with T2D, glucose disposal was decreased after low‐dose insulin. The contribution of the pathological processes that drive NAFLD will differ according to the presence or absence of T2D, and therefore, it is likely that the response to therapy, (especially one that relies on glucose lowering) will also differ.

The changes that we observed in body composition and blood pressure are in line with previous published studies.^32^ On average, patients lost 4.4 kg in weight (4.6%). Despite this, there was no improvement in hepatic steatosis, which is potentially at odds with lifestyle intervention studies in the context of NAFLD.^33^ Furthermore, weight loss did not correlate with change in steatosis as measured by MRS. This may be due to the relatively modest decrease in fat mass and the parallel changes in lean mass and total body water. This contrasts with previous literature on T2DM patients where SGLT2 inhibitors are associated with reductions in total body fat.^34^ Despite a reduction in total body water measured by bioimpedance, there was no significant reduction in hepatic free water content as measured by cT1. Therapies that are able to achieve larger changes, specifically in fat mass, are likely to be more efficacious in the treatment of NAFLD.

In conclusion, we have demonstrated that, in a small cohort of patients without T2D, SGLT2 inhibition was not able to alter the hepatic TAG content or the processes that are crucial to its pathogenesis. We do acknowledge, however, the marked variability in the individual responses of hepatic steatosis to SGLT2 inhibition and that interpretations may be limited by small sample size. Further studies are needed to determine if there is clinical improvement in NAFLD in patients with T2D or impaired glucose handling (fasting or postprandial) and to define the mechanisms by which this might occur. Overall, it seems unlikely that SGLT2 inhibition will be of significant benefit to those patients with NAFLD who have normoglycemia.

## References

[1] Bedogni G , Miglioli L , Masutti F , Tiribelli C , Marchesini G , Bellentani S . Prevalence of and risk factors for nonalcoholic fatty liver disease: the dionysos nutrition and liver study. Hepatology. 2005; 42: 44–52. 15895401 10.1002/hep.20734

[2] Machado M , Marques‐Vidal P , Cortez‐Pinto H . Hepatic histology in obese patients undergoing bariatric surgery. J. Hepatol. 2006; 45: 600–6. 16899321 10.1016/j.jhep.2006.06.013

[3] Ekstedt M , Hagström H , Nasr P *et al* Fibrosis stage is the strongest predictor for disease‐specific mortality in NAFLD after up to 33 years of follow‐up. Hepatology. 2015; 61: 1547–54. 25125077 10.1002/hep.27368

[4] Wong RJ , Cheung R , Ahmed A . Nonalcoholic steatohepatitis is the most rapidly growing indication for liver transplantation in patients with hepatocellular carcinoma in the U.S. Hepatology. 2014; 59: 2188–95. 24375711 10.1002/hep.26986

[5] McPherson S , Hardy T , Henderson E , Burt AD , Day CP , Anstee QM . Evidence of NAFLD progression from steatosis to fibrosing‐steatohepatitis using paired biopsies: implications for prognosis and clinical management. J. Hepatol. 2015; 62: 1148–55. 25477264 10.1016/j.jhep.2014.11.034

[6] Hossain N , Afendy A , Stepanova M *et al* Independent predictors of fibrosis in patients with nonalcoholic fatty liver disease. Clin. Gastroenterol. Hepatol. 2009; 7: 1224–9 1229.e1‐2. 19559819 10.1016/j.cgh.2009.06.007

[7] Armstrong MJ , Hazlehurst JM , Hull D *et al* Abdominal subcutaneous adipose tissue insulin resistance and lipolysis in patients with non‐alcoholic steatohepatitis. Diabetes Obes. Metab. 2014; 16: 651–60. 24962805 10.1111/dom.12272 PMC4190688

[8] Diraison F , Moulin P , Beylot M . Contribution of hepatic de novo lipogenesis and reesterification of plasma non esterified fatty acids to plasma triglyceride synthesis during non‐alcoholic fatty liver disease. Diabetes Metab. 2003; 29: 478–85. 14631324 10.1016/s1262-3636(07)70061-7

[9] Donnelly KL , Smith CI , Schwarzenberg SJ , Jessurun J , Boldt MD , Parks EJ . Sources of fatty acids stored in liver and secreted via lipoproteins in patients with nonalcoholic fatty liver disease. J. Clin. Invest. 2005; 115: 1343–51. 15864352 10.1172/JCI23621 PMC1087172

[10] Stiede K , Miao W , Blanchette HS *et al* Acetyl‐coenzyme A carboxylase inhibition reduces de novo lipogenesis in overweight male subjects: a randomized, double‐blind, crossover study. Hepatology. 2017; 66: 324–34. 28470676 10.1002/hep.29246 PMC5599970

[11] Bailey CJ , Gross JL , Pieters A , Bastien A , List JF . Effect of dapagliflozin in patients with type 2 diabetes who have inadequate glycaemic control with metformin: a randomised, double‐blind, placebo‐controlled trial. Lancet (London, England). 2010; 375: 2223–33. 10.1016/S0140-6736(10)60407-2 20609968

[12] Cefalu WT , Leiter LA , Yoon K‐HH *et al* Efficacy and safety of canagliflozin versus glimepiride in patients with type 2 diabetes inadequately controlled with metformin (CANTATA‐SU): 52 week results from a randomised, double‐blind, phase 3 non‐inferiority trial. Lancet (London, England). 2013; 382: 941–50. 10.1016/S0140-6736(13)60683-2 23850055

[13] Zinman B , Wanner C , Lachin JM *et al* Empagliflozin, cardiovascular outcomes, and mortality in type 2 diabetes. N. Engl. J. Med. 2015; 373: 2117–28. 26378978 10.1056/NEJMoa1504720

[14] Hayashizaki‐Someya Y , Kurosaki E , Takasu T *et al* Ipragliflozin, an SGLT2 inhibitor, exhibits a prophylactic effect on hepatic steatosis and fibrosis induced by choline‐deficient l‐amino acid‐defined diet in rats. Eur. J. Pharmacol. 2015; 754: 19–24. 25701721 10.1016/j.ejphar.2015.02.009

[15] Tahara A , Kurosaki E , Yokono M *et al* Effects of SGLT2 selective inhibitor ipragliflozin on hyperglycemia, hyperlipidemia, hepatic steatosis, oxidative stress, inflammation, and obesity in type 2 diabetic mice. Eur. J. Pharmacol. 2013; 715: 246–55. 23707905 10.1016/j.ejphar.2013.05.014

[16] Kuchay MS , Krishan S , Mishra SK *et al* Effect of empagliflozin on liver fat in patients with type 2 diabetes and nonalcoholic fatty liver disease: a randomized controlled trial (E‐LIFT trial). Diabetes Care. 2018; 41: 1801–8. 29895557 10.2337/dc18-0165

[17] Latva‐Rasku A , Honka M‐J , Kullberg J *et al* The SGLT2 inhibitor dapagliflozin reduces liver fat but does not affect tissue insulin sensitivity: a randomized, double‐blind, placebo controlled study with 8‐week treatment in type 2 diabetes patients. Diabetes Care. 2019; 42: 931–7. 30885955 10.2337/dc18-1569

[18] Cusi K , Bril F , Barb D *et al* Effect of canagliflozin treatment on hepatic triglyceride content and glucose metabolism in patients with type 2 diabetes. Diabetes Obes. Metab. 2019; 21: 812–21. 30447037 10.1111/dom.13584

[19] Shimizu M , Suzuki K , Kato K *et al* Evaluation of the effects of dapagliflozin, a sodium‐glucose co‐transporter‐2 inhibitor, on hepatic steatosis and fibrosis using transient elastography in patients with type 2 diabetes and non‐alcoholic fatty liver disease. Diabetes Obes. Metab. 2019; 21: 285–92. 30178600 10.1111/dom.13520

[20] Akuta N , Kawamura Y , Watanabe C *et al* Impact of SGLT2 inhibitor to histological features and glucose metabolism of non‐alcoholic fatty liver disease complicated by diabetes mellitus. Hepatol. Res. 2018; 49: 531‐–9. 10.1111/hepr.13304 30577089

[21] Banerjee R , Pavlides M , Tunnicliffe EM *et al* Multiparametric magnetic resonance for the non‐invasive diagnosis of liver disease. J. Hepatol. 2014; 60: 69–77. 24036007 10.1016/j.jhep.2013.09.002 PMC3865797

[22] Szczepaniak LS , Nurenberg P , Leonard D *et al* Magnetic resonance spectroscopy to measure hepatic triglyceride content: prevalence of hepatic steatosis in the general population. Am. J. Physiol. Endocrinol. Metab. 2005; 288: E462–8. 15339742 10.1152/ajpendo.00064.2004

[23] Steele R . Use of C14‐glucose to measure hepatic glucose production following an intravenous glucose load or after injection of insulin. Metabolism. 1959; 8: 512–19. 13666397

[24] Saponaro C , Pattou F , Bonner C . SGLT2 inhibition and glucagon secretion in humans. Diabetes Metab. 2018; 44: 383–5. 30017776 10.1016/j.diabet.2018.06.005

[25] Bolinder J , Ljunggren Ö , Kullberg J *et al* Effects of dapagliflozin on body weight, total fat mass, and regional adipose tissue distribution in patients with type 2 diabetes mellitus with inadequate glycemic control on metformin. J. Clin. Endocrinol. Metab. 2012; 97: 1020–31. 22238392 10.1210/jc.2011-2260

[26] Sattar N , Fitchett D , Hantel S , George JT , Zinman B . Empagliflozin is associated with improvements in liver enzymes potentially consistent with reductions in liver fat: results from randomised trials including the EMPA‐REG OUTCOME® trial. Diabetologia. 2018; 61: 2155–63. 30066148 10.1007/s00125-018-4702-3 PMC6133166

[27] Dowman JK , Tomlinson JW , Newsome PN . Systematic review: the diagnosis and staging of non‐alcoholic fatty liver disease and non‐alcoholic steatohepatitis. Aliment. Pharmacol. Ther. 2011; 33: 525–40. 21198708 10.1111/j.1365-2036.2010.04556.x PMC3080668

[28] Santhekadur PK , Kumar DP , Sanyal AJ . Preclinical models of non‐alcoholic fatty liver disease. J. Hepatol. 2018; 68: 230–7. 29128391 10.1016/j.jhep.2017.10.031 PMC5775040

[29] Sanyal AJ , Chalasani N , Kowdley KV *et al* Pioglitazone, vitamin E, or placebo for nonalcoholic steatohepatitis. N. Engl. J. Med. 2010; 362: 1675–85. 20427778 10.1056/NEJMoa0907929 PMC2928471

[30] Merovci A , Solis‐Herrera C , Daniele G *et al* Dapagliflozin improves muscle insulin sensitivity but enhances endogenous glucose production. J. Clin. Invest. 2014; 124: 509–14. 24463448 10.1172/JCI70704 PMC3904617

[31] Ahn CH , Oh TJ , Kwak SH , Cho YM . Sodium‐glucose cotransporter‐2 inhibition improves incretin sensitivity of pancreatic β‐cells in people with type 2 diabetes. Diabetes Obes. Metab. 2018; 20: 370–7. 28786557 10.1111/dom.13081

[32] Rajeev SP , Cuthbertson DJ , Wilding JPH . Energy balance and metabolic changes with sodium‐glucose co‐transporter 2 inhibition. Diabetes Obes. Metab. 2016; 18: 125–34. 26403227 10.1111/dom.12578

[33] Kenneally S , Sier JH , Moore JB . Efficacy of dietary and physical activity intervention in non‐alcoholic fatty liver disease: a systematic review. BMJ Open Gastroenterol. 2017; 4: e000139. 10.1136/bmjgast-2017-000139 PMC5508801 28761689

[34] Tobita H , Sato S , Miyake T , Ishihara S , Kinoshita Y . Effects of dapagliflozin on body composition and liver tests in patients with nonalcoholic steatohepatitis associated with type 2 diabetes mellitus: a prospective, open‐label, uncontrolled study. Curr. Ther. Res. Clin. Exp. 2017; 87: 13–19. 28912902 10.1016/j.curtheres.2017.07.002 PMC5587885

